# Left atrial strain parameters derived by echocardiography are impaired in patients with acute myocarditis and preserved systolic left ventricular function

**DOI:** 10.1007/s10554-023-02827-9

**Published:** 2023-03-24

**Authors:** Christine Meindl, Michael Paulus, Florian Poschenrieder, Okka W. Hamer, Florian Zeman, Lars S. Maier, Kurt Debl

**Affiliations:** 1 Department of Internal Medicine II, University Hospital Regensburg, 93053 Regensburg, Germany; 2 Institute of Radiology, University Hospital Regensburg, Regensburg, Germany; 3 Department of Pneumology, Donaustauf Hospital, Donaustauf, Germany; 4 Center for Clinical Studies, University Hospital Regensburg, Regensburg, Germany

**Keywords:** Myocarditis, Left atrial strain imaging, Speckle tracking, Diastolic dysfunction

## Abstract

**Purpose**: Data derived by cardiac magnetic resonance (CMR) feature tracking suggest that not only left ventricular but also left atrial function is impaired in patients with acute myocarditis. Therefore, we investigated the diagnostic value of speckle tracking echocardiography of the left ventricle and left atrium in patients with acute myocarditis and normal left ventricular ejection fraction (LVEF). **Methods and results**: 30 patients with acute myocarditis confirmed by CMR according to the Lake Louise criteria and 20 healthy controls were analyzed including global longitudinal strain (GLS) and left atrial (LA) strain parameters. Although preserved LVEF was present in both groups, GLS was significantly lower in patients with acute myocarditis (GLS − 19.1 ± 1.8% vs. GLS − 22.1 ± 1.7%, p < 0.001). Further diastolic dysfunction measured by E/e’ mean was significantly deteriorated in the myocarditis group compared to the control group (E/e’ mean 6.4 ± 1.6 vs. 5.5 ± 1.0, p = 0.038). LA reservoir function (47.6 ± 10.4% vs. 55.5 ± 10.8%, p = 0.013) and LA conduit function (-33.0 ± 9.6% vs. -39.4 ± 9.5%, p = 0.024) were significantly reduced in patients with acute myocarditis compared to healthy controls. Also left atrial stiffness index (0.15 ± 0.05 vs. 0.10 ± 0.03, p = 0.003) as well as left atrial filling index (1.67 ± 0.47 vs. 1.29 ± 0.34, p = 0.004) were deteriorated in patients with myocarditis compared to the control group. **Conclusion**: In patients with acute myocarditis and preserved LVEF not only GLS but also LA reservoir function, LA conduit function and left atrial stiffness index as well as left atrial filling index were impaired compared to healthy controls indicating ventricular diastolic dysfunction and elevated LV filling pressures.

## Introduction

The contribution of the left atrium (LA) to cardiac hemodynamics comprises the modulation of left ventricular filling by the interaction of atrial reservoir, conduit and booster contractile function. The function of LA has been characterized by pressure-volume curves via invasive examination [1, 2].

It is known that the LA reacts in a very sensitive way to ongoing volume and pressure overload secondary to elevated left ventricular filling pressures [3]. More recently speckle tracking echocardiography – a well validated tool for the assessment of the left ventricular (LV) function – has been implemented for the assessment of regional and global LA function [4, 5].

Nochioka et al. showed that in patients with cardiac amyloidosis all LA function phases including reservoir, conduit and booster function were impaired compared to healthy controls. The results highly correlated with LV deformation [6].

Furthermore, Matsubara et al. compared 28 children with “multisystem inflammatory syndrome in children (MIS-C)” due to Coronavirus disease 2019 (COVID-19) infection with 20 healthy controls and 20 consecutive patients with Kawasaki disease (KD) [7]. In patients with MIS-C, LV systolic and diastolic function measured by deformation parameters were impaired in contrast to healthy subjects and KD patients. MIS-C patients with myocardial injury were more affected than those without. Peak left atrial strain was one of the strongest parameters to predict myocardial injury in MIS-C besides global longitudinal strain (GLS) and others. Interestingly a good recovery of systolic function was observed while diastolic dysfunction persisted during an abbreviated follow-up [7].

In a multicenter study including 322 patients with different cardiovascular diseases, Inoue et al. investigated LA strain and other echocardiographic parameters and compared them with invasively measured LV filling pressures [8]. GLS and LV filling pressures turned out to be the strongest determinants of LA reservoir and pump strain. In patients with reduced LV systolic function, LA strain parameters were able to identify increased LV filling pressures most accurately. In addition, high values of LA pump strain showed good accuracy in identifying normal LV filling pressures in patients with preserved systolic function [8].

Only scarce data exist concerning LA strain parameters in adult patients with acute myocarditis. Dick et al. investigated 30 patients with feature tracking by cardiac magnetic resonance (CMR) imaging. In this study, a decreased LA reservoir and conduit function in patients with acute myocarditis compared to healthy controls was detected while LA booster pump function was preserved [9].

Doerner et al. analyzed 86 patients with acute myocarditis and 30 healthy controls with feature tracking derived strain analysis by CMR. LA conduit function was reduced in patients with acute myocarditis compared to healthy controls. Furthermore, LA conduit function represented one of the best independent predictors of myocarditis in patients with preserved LV function [10].

These data derived by CMR feature tracking suggest that not only left ventricular but also left atrial function is impaired in patients with acute myocarditis. However, data on echocardiographic left atrial strain analysis in patients with acute myocarditis are lacking. Therefore, we investigated the diagnostic value of speckle tracking echocardiography of the left ventricle and left atrium in patients with acute myocarditis and normal left ventricular ejection fraction (LVEF).

## Methods

### Study population

As described in a previous study of our working group we retrospectively identified 31 patients with acute myocarditis and preserved LVEF admitted to the University Hospital Regensburg between November 2015 and November 2019 [11]. The diagnosis of myocarditis was confirmed by typical clinical presentation and by typical findings in CMR. The so-called typical clinical presentation comprises “infarct-like” myocarditis including chest pain, elevation of troponin and creatine kinase levels, ECG abnormalities and preceding signs of infection. In 30 patients left ventricular as well as left atrial strain parameters could be investigated.

CMR and transthoracic echocardiography (TTE) were conducted in the acute phase of myocarditis. Follow up TTE were performed after a median of 14 weeks (10–24). Complete follow-ups were present in 20 cases. Patients with a history of severe symptoms e.g. sudden cardiac death, heart failure or reduced LVEF were excluded. In addition, patients aged < 18 years and patients with poor TTE image quality were not included. Clinical data and blood samples of all participants were analyzed. The study was approved by the local ethics committee.

20 healthy age- and gender-matched subjects were recruited to serve as a control group.

The control group did not have a history of myocarditis and other cardiac diseases. All volunteers gave written informed consent and were investigated by complete TTE.

No external funding was obtained to support the study [11].

### Echocardiography

As previously described[11] TTE was performed in the first few days of acute myocarditis using IE33 and Epiq CVx (Philips Medical Systems, Amsterdam, The Netherlands) ultrasound systems as well as S5-1 or X5-1 (Philips Medical Systems, Amsterdam, The Netherlands) transducers. Routine two-dimensional (2D) cine loops were obtained and three apical views (four-chamber, two-chamber and three-chamber view) were stored digitally. LVEF was calculated by a biplane Simpson’s method from apical four- and two-chamber views. TTE were analyzed offline using the IntelliSpace QLAB software (Philips Medical Systems, Amsterdam, The Netherlands) by two experienced observers (C.M., K.D.). TTE were studied independently by each observer.

### Speckle tracking analysis

Speckle tracking analysis was performed on a frame-by-frame basis by automatic tracking of acoustic markers (speckles) throughout the cardiac cycle. The duration of systole was defined in the apical three-chamber view by marking the aortic valve opening and closure. The myocardial borders were traced from the apical four-, two- and three-chamber views in the end-systolic frame of the 2D images in order to analyze global longitudinal strain (GLS).

In accordance with the consensus document of the EACVI/ASE/Industry the region of interest (ROI) was defined by the endocardial border which symbolizes the inner contour of the myocardium [12]. If necessary, endocardial borderlines were manually adapted and motion tracking was performed automatically by the IntelliSpace QLAB software.

Peak systolic strain was determined as the maximum value of the peak negative strain during systole. By assessing the peak systolic longitudinal strain in all 17 longitudinal segments, global longitudinal strain was provided by the software as the average value of the different segments.

**Fig. 1:**
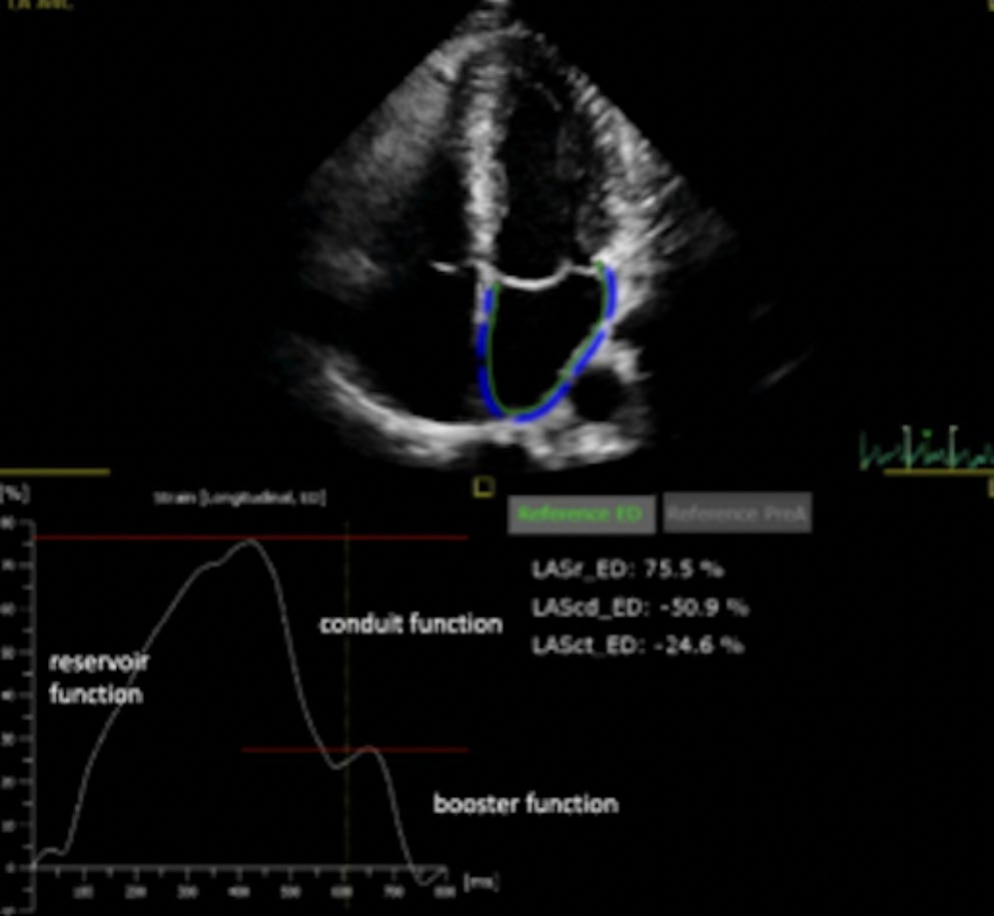
Example of left atrial strain imaging including three phases: LA reservoir (LASr), LA conduit (LAScd) and LA booster function (LASct)

LA volumes and areas were received by measurements in apical four- and two-chamber views. As commonly described the pulmonary veins, LA appendage and mitral leaflets were excluded for LA analysis [13]. In order to determine LA volume index (LAVI) LA volumes were indexed to body surface area (BSA). Speckle tracking analysis of the LA was conducted in the apical four- and two-chamber views. The endocardial surface was tracked automatically by the software (IntelliSpace QLAB, Philips Medical Systems, Amsterdam, The Netherlands) and, if necessary, endocardial borderlines were manually adapted. The analysis of the apical four- and two-chamber view provided 6 segments per view resulting in a global 12 segment-model of the LA. As previously described the onset of the QRS complex was used as a reference point of strain measurements using the so called R-R gating model [13]. Fig. 1 depicts the different phases of LA hemodynamics: LA reservoir function (PALS: peak atrial longitudinal strain), LA conduit function (LACS: LA strain during conduit phase) and LA booster or contractile function (PACS: peak atrial contraction strain). In addition LA stiffness index which represents the ratio of E/e’ mean and LA reservoir function as well as LA filling index which symbolizes the ratio of E wave velocity and LA reservoir function were calculated[14].

### Cardiac magnetic resonance imaging

As previously described[11] CMR was performed in the first few days of acute myocarditis on a 1.5 or 3 Tesla scanner (Magnetom Avanto or Magnetom Skyra, Siemens Healthcare, Erlangen, Germany) using a 32-channel phased-array receiver coil. CMR images were acquired during breath hold and with ECG-gating. The CMR imaging protocol included short axis fat-saturated T2-weighted sequences searching for myocardial edema and postcontrast late gadolinium-enhanced T1-weighted phase-sensitive inversion recovery (PSIR) sequences in short axis, vertical long axis and horizontal long axis view for the detection of myocardial injury (LGE). According to the updated Lake Louise criteria we used T2-weighted images in order to search for edema and quantified LGE to detect non-ischemic myocardial injury [15].

### Statistical analysis

Statistical analysis was performed using SPSS Version 25 (International Business Machines Corporation, IBM, Armonk, NY USA). Categorical data are presented as absolute and relative frequencies whereas continuous variables with normal distribution are presented as mean ± standard deviation, variables with skewed distribution as median (interquartile range, IQR). Differences between continuous variables in paired data were tested with a paired t-test, continuous variables in unpaired data were compared with an unpaired t-test. Differences in nominal variables and dichotomous variables in unpaired data were evaluated by Pearson’s chi-squared test and Fisher’s exact test, respectively. A *p-*value of < 0.05 was considered statistically significant.

## Results

Baseline characteristics of the study population are depicted in Table 1. Mean age of patients with acute myocarditis was 29 ± 9 years compared to 33 ± 4 years in the control group (p = 0.049). No significant differences were revealed concerning gender or comorbidities like arterial hypertension or valvular heart disease. BMI levels were significantly lower in the control group than in patients with myocarditis (BMI 23.7 ± 2.4 kg/m^2^ vs. 25.9 ± 3.6 kg/m^2^, p = 0.021). 83.3% of patients with acute myocarditis had preceding signs of infection and in 76.7% ECG abnormalities were present. Coronary angiography was performed in 50% of patients (n = 15) with myocarditis and obstructive coronary artery disease was excluded in all patients. In 10 cases LV filling pressures were measured invasively. The median LV filling pressure was 22 ± 6 mmHg, indicating elevated LV filling pressures.

**Table 1:** Clinical and echocardiographic characteristics of the myocarditis and control group

	Myocarditis (n = 30)	Control (n = 20)	p-value
Age, years	29 ± 9	33 ± 4	**0.049**
Female gender	2 (6.7)	1 (5.0)	1.000
BMI, kg/m²	25.9 ± 3.6	23.7 ± 2.4	**0.021**
Arterial hypertension	1 (3.3)	0	1.000
Hyperlipidemia	1 (3.3)	0	1.000
Diabetes mellitus	0	0	
Valvular heart disease	1 (3.3)	0	1.000
ECG abnormalities	23 (76.7)		
Preceding signs of infection	25 (83.3)		
Coronary angiography performed	15 (50.0)		
Left ventricular end diastolic pressure (n = 10), mmHg	22 ± 6		
Diagnosis of coronary artery disease	1 (3.3)		
LVEF, %	58 ± 4	60 ± 4	0.117
Global longitudinal strain, %	-19.1 ± 1.8	-22.1 ± 1.7	**< 0.001**
E, cm/s	76 ± 18	70 ± 14	0.192
E/A	1.4 ± 0.4	1.5 ± 0.3	0.633
e‘ septal, cm/s	11.1 ± 2.9	10.9 ± 2.4	0.852
e‘ lateral, cm/s	13.1 ± 2.8	15.8 ± 2.4	**0.001**
E/e‘	6.4 ± 1.6	5.5 ± 1.0	**0.038**
IVSd, mm	10 ± 2	9 ± 2	**0.043**
PWd, mm	10 ± 1	9 ± 1	**0.008**
LVESD, mm	35 ± 6	34 ± 4	0.511
LVEDD, mm	49 ± 6	48 ± 3	0.600
LV mass, g	155 ± 46	129 ± 38	**0.037**
RVEDD, mm	33 ± 5	34 ± 2	0.308
LAVI, ml/m²	25 ± 9	23 ± 6	0.227
LA area two chamber, cm²	16 ± 4	14 ± 3	0.171
LA area four chamber, cm²	17 ± 4	17 ± 3	0.906
LASr, %	47.6 ± 10.4	55.5 ± 10.8	**0.013**
LA conduit function, %	-33.0 ± 9.6	-39.4 ± 9.5	**0.024**
LA booster function, %	-14.6 ± 5.9	-16.1 ± 4.9	0.364
LA stiffness index	0.15 ± 0.05	0.10 ± 0.03	**0.003**
LA filling index	1.67 ± 0.47	1.29 ± 0.34	**0.004**
LAVI/LA reservoir function	0.48 (0.40–0.62)	0.41 (0.31–0.51)	**0.030**
peak Troponin T, pg/ml	8466 (1458–26,825)		
peak CRP, mg/l	35.3 (10.0–86.0)		
peak NTproBNP, pg/ml	393 (211–632)		

Continuous variables with normal distribution are expressed as mean ± SD, variables with skewed distribution as median (IQR). Categorical variables are expressed as n (%)

BMI: body mass index; ECG: electrocardiogram; LVEF: left ventricular ejection fraction; PWd: posterior wall thickness; IVSd: interventriculuar septal wall thickness; LVESD: left ventricular end-systolic diameter; LVEDD: left ventricular end-diastolic diameter; RVEDD: right ventricular end-diastolic diameter; LAVI: left atrial volume index; LA: left atrium; LASr LA reservoir function; CRP: C reactive protein; NT proBNP: N-terminal prohormone of brain natriuretic peptide

Peak troponin and creatine kinase levels were selected to present the maximum extent of myocardial injury. Median peak troponin level was 8466 pg/ml (1458–26,825) and median maximum C reactive protein (CRP) was 35.3 mg/l (10.0–86.0) in patients with acute myocarditis.

Ejection fraction was preserved in the myocarditis group (LVEF 58 ± 4%) as well as in the control group (LVEF 60 ± 4%, p = 0.117, Table 1). Diastolic posterior (PWd) and septal wall thickness (IVSd) measured by echocardiography was significantly higher in patients with myocarditis than in the control group (PWd 10 ± 1 vs. 9 ± 1 mm, p = 0.008; IVSd 10 ± 2 vs. 9 ± 2 mm, p = 0.043). Furthermore, diastolic dysfunction measured by E/e’ mean was significantly deteriorated in the myocarditis group compared to the control group (6.4 ± 1.6 vs. 5.5 ± 1.0, p = 0.038). Pulsed-wave Tissue Doppler Imaging (PW-TDI) of the lateral LV wall revealed lower values in the myocarditis than in the control group (Table 1).

**Fig. 2:**
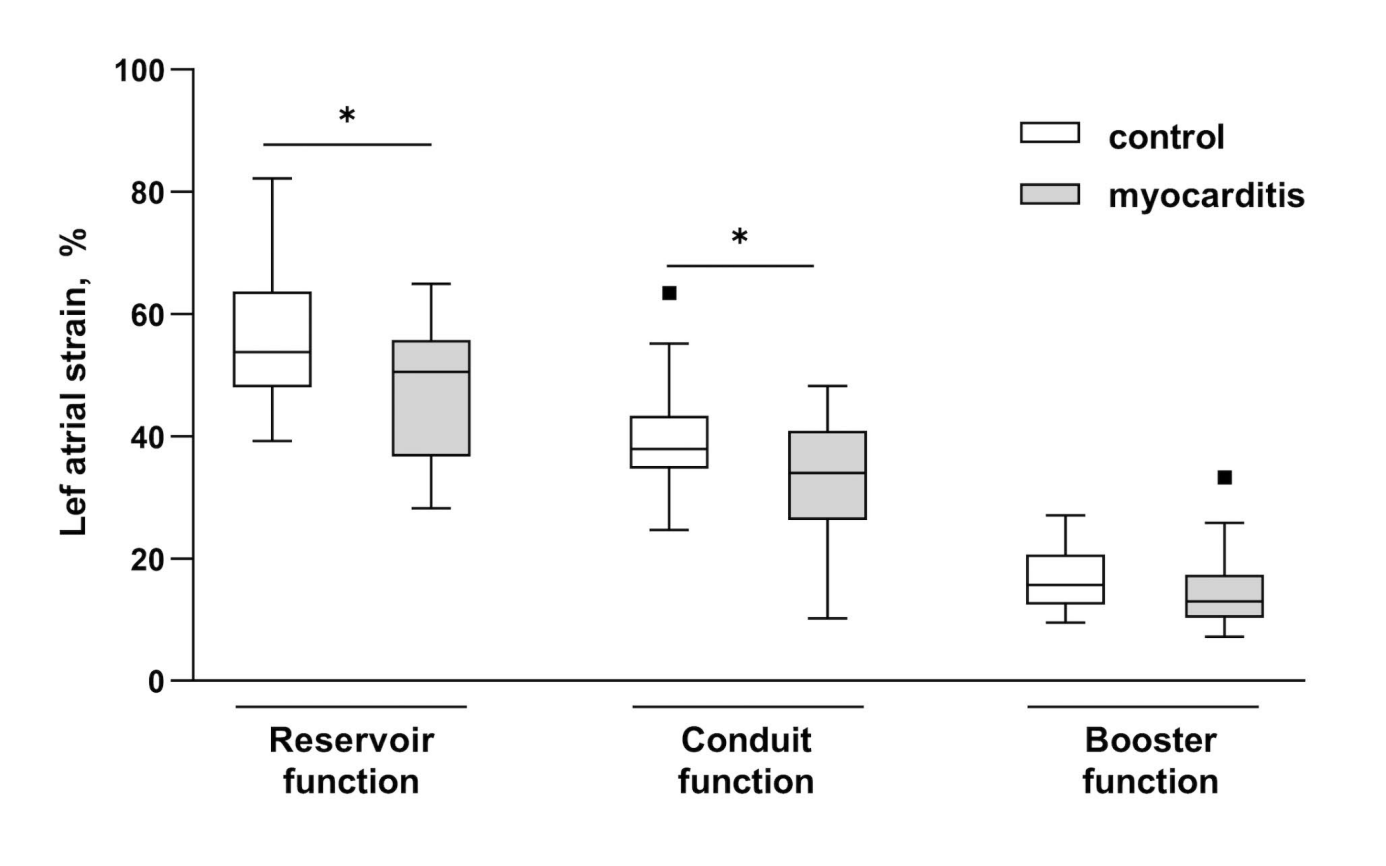
**Left atrial strain parameters in patients with myocarditis and in the control group.** Data are shown as Tukey-style boxplots. * p < 0.05, ** p < 0.01 in t-test

**Fig. 3:**
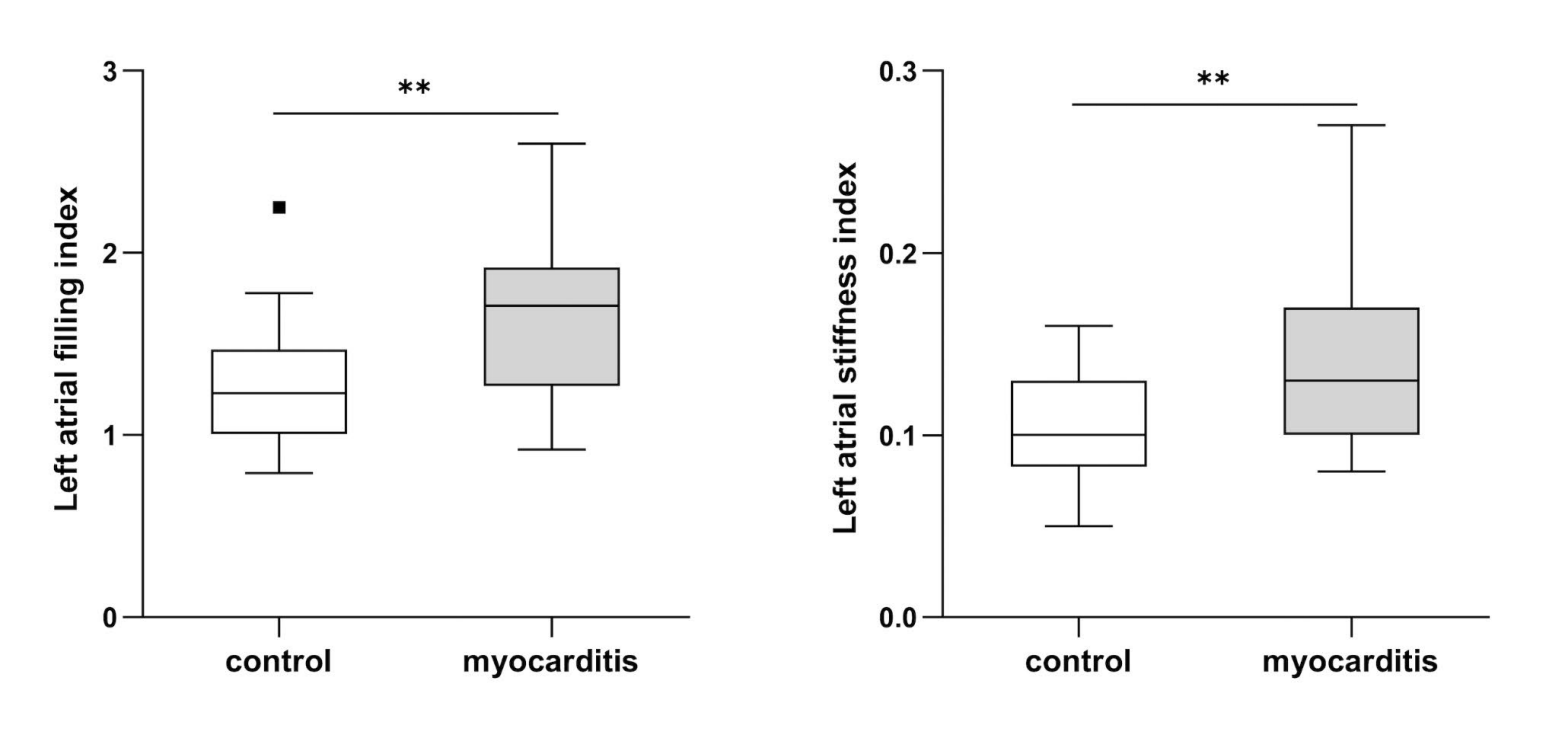
**Left atrial strain indices in the myocarditis and control group.** Data are shown as Tukey-style boxplots. ** p < 0.01 in t-test

Despite preserved ejection fraction in both groups GLS was significantly decreased in patients with acute myocarditis (GLS − 19.1 ± 1.8%) compared to the control group (GLS − 22.1 ± 1.7%, p < 0.001). No differences concerning indexed left atrial volumes (LAVI) or LA area were revealed. However, LA reservoir function was impaired in patients with myocarditis compared to healthy controls (47.6 ± 10.4% vs. 55.5 ± 10.8%, p = 0.013, Fig. 2). Also, LA conduit function was deteriorated in the myocarditis compared to the control group (-33.0 ± 9.6% vs. -39.4 ± 9.5%, p = 0.024). Further significant differences were detected between both groups concerning LA stiffness index (Fig. 3). In addition LA filling index revealed significantly higher values in patients with myocarditis compared to healthy controls (Fig. 3). As depicted in Table 1 the ratio of LAVI and LA reservoir function showed significantly higher values in patients with myocarditis than in the control group.

Table 2 depicts the echocardiographic characteristics of patients with myocarditis at baseline and at a three months follow-up. Follow-up was available in 20 patients. GLS improved indicating significantly higher values at follow-up compared to baseline (-21.8 ± 2.5% vs. -19.1 ± 1.8, p = 0.002). Further E/e’ mean values of the myocarditis group ameliorated at follow-up (6.4 ± 1.6 vs. 5.5 ± 1.0, p = 0.038).

**Table 2:** Echocardiographic characteristics in patients with myocarditis at baseline and follow-up (n = 20)

	Baseline	Follow-up	p-value
LVEF, %	57 ± 3	60 ± 5	0.086
Global longitudinal strain, %	-19.1 ± 1.8	-21.8 ± 2.5	**0.002**
E, cm/s	75 ± 18	71 ± 12	0.366
E/A	1.5 ± 0.4	1.4 ± 0.3	0.400
e‘ septal, cm/s	11.6 ± 3.0	12.1 ± 4.1	0.429
e‘ lateral, cm/s	13.7 ± 3.1	14.2 ± 4.5	0.654
E/e‘	6.4 ± 1.6	5.5 ± 1.0	**0.038**
IVSd, mm	10 ± 2	9 ± 2	0.248
PWd, mm	10 ± 1	9 ± 2	0.181
LVESD, mm	34 ± 4	37 ± 5	0.126
LVEDD, mm	49 ± 4	49 ± 4	0.645
LV mass, g	155 ± 44	144 ± 40	0.160
RVEDD, mm	33 ± 5	34 ± 4	0.754
LAVI, ml/m²	25 ± 9	25 ± 6	0.792
LA area two chamber, cm²	17 ± 5	17 ± 4	0.805
LA area four chamber, cm²	17 ± 4	17 ± 3	0.533
LASr, %	48.3 ± 11.0	49.9 ± 8.1	0.487
LA conduit function, %	-32.7 ± 10.4	-35.0 ± 9.4	0.225
LA booster function, %	-15.6 ± 6.5	-15.0 ± 6.0	0.704
LA stiffness index	0.15 ± 0.07	0.12 ± 0.04	0.080
LA filling index	1.68 ± 0.56	1.47 ± 0.24	0.153
LAVI/LA reservoir function	0.51 (0.40–0.62)	0.54 (0.35–0.62)	0.350

Variables with normal distribution are expressed as mean ± SD, variables with skewed distribution as median (IQR). Median time from baseline to follow-up was 14 (10–24) weeks

LASr LA reservoir function

## Discussion

In the present study we focused on patients with acute myocarditis and preserved ejection fraction. The major findings of this study are:

- In patients with acute myocarditis, LA reservoir function, LA conduit function and LA stiffness index as well as LA filling index were impaired compared to healthy controls, indicating elevated filling pressure and diastolic dysfunction.
- E/e’ mean values were higher and e’ lateral was lower in the myocarditis than in the control group, which might be caused by myocardial injury of the lateral left ventricular wall.
- Despite normal LVEF values, GLS was significantly reduced in the myocarditis group compared to a healthy control group suggesting subtle pathologies.

Dick et al. demonstrated for the first time the feasibility of CMR feature tracking of the left atrium in patients with acute myocarditis [9]. This study revealed an impaired LA reservoir and conduit function in patients with myocarditis compared to a healthy control group. In contrast the active booster pump function of the left atrium was preserved [9]. In the present study we could confirm these results of a CMR study in an echocardiography-based investigation. To our knowledge we show for the first time that LA reservoir and conduit function as well as LA stiffness index as well as LA filling index derived by echocardiography is impaired in adult patients with acute myocarditis despite preserved left ventricular function.

It is known that the LA contributes to a dynamic modulation of left ventricular filling and that the LA is not a passive heart chamber. The pathophysiological influence of the different LA functions i.e. reservoir, conduit and booster pump function is not fully understood so far. LA reservoir function is supposed to reflect LA compliance and LA active relaxation and may serve as a compensatory mechanism of congestive left ventricular failure at early stages. Contrarily, LA conduit function represents the extent of LA compliance and is already affected by diastolic left ventricular dysfunction at early stages. The third phase of LA function – LA booster pump- symbolizes LA contraction and influences ventricular filling and cardiac output [16–19].

There is evidence that LA reservoir function correlates with LV filling pressures [18, 20]. This fact could explain that in our study, LA reservoir but not LA booster pump function was impaired in patients with acute myocarditis compared to healthy controls. Invasive measurements of LV end diastolic pressure (LVEDP) were available in 10 patients. However, in these cases the median LVEDP was considerably elevated (22 ± 6 mmHg) indicating diastolic dysfunction in patients with myocarditis and preserved systolic LV function.

LA conduit function as a marker of early diastolic abnormalities was also reduced in patients with acute myocarditis compared to healthy controls. This finding could be confirmed as diastolic dysfunction measured by E/e’ mean was significantly deteriorated in the myocarditis group compared to the control group. Pulsed-wave TDI of the lateral LV wall revealed lower values in the myocarditis than in the control group which might be caused by edema and myocardial injury of the lateral left ventricular wall. As previously described, there exist regional differences, with inferior and inferolateral segments being the most affected by myocardial injury in CMR in patients with myocarditis [11].

As described by Nielsen et al. left atrial reservoir and conduit function decrease significantly while ageing [13]. In the present study the control group was older than the patients of the myocarditis group. However left atrial reservoir and conduit function were impaired in the myocarditis group compared to older but healthy subjects indicating diastolic dysfunction and elevated left ventricular filling pressures.

The role of diastolic dysfunction in patients with acute myocarditis has been investigated in several studies and there exists growing evidence that diastolic abnormalities might be important with regard to the long-term outcome of patients with myocarditis [9].

Escher et al. prospectively analyzed 50 patients with acute myocarditis who underwent endomyocardial biopsies. The mean follow-up period was 72 months. At follow-up, 90% of patients had a normal or improved left ventricular function. Despite preserved or normalized ejection fraction, 49% of patients presented with heart failure symptoms 4–6 years after acute myocarditis. These symptoms were caused by diastolic dysfunction [21]. In our study no significant differences concerning left atrial reservoir and conduit function were present at a 3 months follow-up. However, in contrast to the study of Escher et al. [21] patients with severe heart failure symptoms or reduced ejection fraction at baseline were excluded in the present study.

Matsubara et al. investigated 28 children with multisystem inflammatory syndrome in children (MIS-C) associated with COVID-19 and compared the results to healthy children and children with Kawasaki disease [7]. The best parameters in predicting myocardial injury in MIS-C included GLS and peak left atrial strain. Comparably to our study, patients with MIS-C showed subtle changes in strain imaging although LVEF was preserved, suggesting subclinical myocardial injury [7]. The analysis of LA strain parameters in the assessment of diastolic dysfunction turned out to be the strongest single index associated with myocardial injury in the study by Matsubara et al. Therefore, the authors hypothesized that peak left atrial strain may be a good indicator for atrioventricular coupling and LV diastolic dysfunction [7].

Considering the results of our study, we speculate that the extent of myocardial injury may correlate with the persistence of diastolic dysfunction as a marker of structural changes. Further studies are needed to investigate this relationship.

Concerning GLS, Caspar et al. detected myocardial dysfunction by 2D and 3D speckle tracking analysis in patients late after an acute episode of myocarditis and preserved LV function. Mean delay between the diagnosis of acute myocarditis and follow-up echocardiography was 21.7 ± 23.4 months [22]. In the present study, GLS values improved significantly at a three months follow-up. In contrast to our study Caspar et al. [22] investigated patients with a history of myocarditis but the authors did not analyze strain imaging parameters of these patients in the acute phase of myocarditis.

Stöbe et al. revealed myocardial injury in a relatively small cohort of patients with SARS-CoV-2 infection (severe acute respiratory syndrome-coronavirus-2) even in patients with mild symptoms [23]. Despite normal LV-EF, speckle tracking analysis of the left ventricle was abnormal in most cases. Interestingly, the authors observed a specific deformation pattern which was described as a “reverse basal tako-tsubo-like syndrome”. It was speculated that these abnormalities were caused by pronounced edema and/or myocardial injury in the basal LV segments. [23]

In contrast to GLS limited but growing data exist concerning left atrial strain imaging. Therefore deformation analysis by speckle tracking echocardiography of the left ventricle as well as the left atrium should be added to routine diagnostics in patients with suspected acute myocarditis. These parameters should also be applied at the time of follow-up visits. The importance of speckle tracking echocardiography is even more pronounced in institutions with limited access to CMR.

Some limitations of the present study warrant consideration:

Firstly -as already mentioned in a previous study[11]- our study represents a single-center experience with a retrospective study design. Secondly, 10 patients were lost to follow-up which might also have had an impact on the study results. Thirdly, the number of subjects included in our study was relatively small that is why further studies are needed to evaluate our results in a larger study cohort. Fourthly, we did not analyze circumferential and radial strain parameters. We chose to investigate the longitudinal strain parameters as it is known that in patients with acute myocarditis the most affected myocardial layer is the subepicardium and there the fibers are mainly directed in a longitudinal way [22, 24]. Furthermore, longitudinal strain is the most robust parameter concerning speckle tracking. The study might also have revealed additional information if we had used circumferential or radial strain in order to find out more about the myocardial fiber architecture.

## Conclusion

In patients with acute myocarditis and preserved LVEF, not only GLS but also LA reservoir function, LA conduit function and left atrial stiffness index as well as LA filling index were impaired compared to healthy controls, indicating ventricular diastolic dysfunction and elevated LV filling pressures. Due to growing evidence left atrial and left ventricular strain imaging should be added to routine diagnostics in patients with suspected acute myocarditis.

## Data Availability

The data underlying this article will be shared on reasonable request to the corresponding author.
